# Rotifers from selected inland saline waters in the Chihuahuan Desert of México

**DOI:** 10.1186/1746-1448-4-7

**Published:** 2008-06-04

**Authors:** Elizabeth J Walsh, Thomas Schröder, Robert L Wallace, Judith V Ríos-Arana, Roberto Rico-Martínez

**Affiliations:** 1 Department of Biological Sciences, University of Texas – El Paso, El Paso, TX 79968, USA; 2 Department of Biology, Ripon College, Ripon, WI 54971, USA; 3 Instituto de Ingeniería y Tecnología, Universidad Autónoma de Cd. Juárez, Chihuahua, México; 4 Departamento de Química, Universidad Autónoma de Aguascalientes, Aguascalientes, México

## Abstract

**Background:**

In spite of considerable efforts over past decades we still know relatively little regarding the biogeography of rotifers of inland waters in México. To help rectify this we undertook an extensive survey of the rotifer fauna of 48 water bodies in the Chihuahuan Desert of México.

**Results:**

Of the sites surveyed, 21 had salinities ≥ 2000 μS cm^-1 ^and in these we found 57 species of monogonont rotifers and several bdelloids. Species richness in the saline sites varied widely, with a range in species richness of 1 to 27 and a mean (± 1SD) = 8.8 (± 6.2). Collectively all sites possess relatively high percent single- and doubletons, 33.3 and 21.7%, respectively. Simpson's Asymmetric Index indicated that similarity in rotifer species composition varied widely among a set of 10 sites. These were selected because they were sampled more frequently or represent unusual habitats. These SAI values ranged from 0.00 (complete dissimilarity) to 1.00 (complete similarity). The Jaccard Index varied between 0.00 and 0.35. This observation probably reflects similarities and differences in water chemistry among these sites. Inland saline systems differed in their chemical composition by region. Conductivity was related to hardness and alkalinity. In addition, hardness was positively associated with chloride and sulfate. RDA showed that several species were positively associated with chloride concentration. Other factors that were significantly associated with rotifer species included the presence of macrophytes, nitrate content, oxygen concentration, TDS, latitude and whether the habitat was a large lake or reservoir.

**Conclusion:**

This study illustrates the diversity of the rotiferan fauna of inland saline systems and the uniqueness among waterbodies. Conservation of these systems is needed to preserve these unique sources of biodiversity that include rotifers and the other endemic species found in association with them.

## Background

Rotifers are widely recognized as being important components of freshwater ecosystems, and whether this assessment is based on numbers or biomass, their contribution to trophic dynamics in these waters is striking. In some instances their importance even exceeds that of the microcrustaceans: cladocerans and copepods [1]. In estuarine and marine habitats, rotifers are generally thought to play a minor role in community dynamics [2-5]. Therefore brackish and marine rotifers, with the notable exception of the *Brachionus plicatilis *species complex, have received little attention worldwide. Because of its value in aquaculture [6-8], this species complex has received special attention and this intense study has yielded valuable insights into evolutionary processes such as cryptic speciation and molecular phylogenetics [9-12] and genomics [13]. In addition, rotifer species inhabiting saline and subsaline lakes in northern Canada possessed greater haplotype diversity than their freshwater counterparts [14].

While quite a bit is known about zooplankton present in México (e.g., copepods [15,16]; cladocerans [17], few reports have been published on brackish and marine rotifers [3,18,19]. Although some of these studies have focused on species diversity and community dynamics [4], none of them have included the Chihuahuan Desert.

With increased exploitation of aquifers for agriculture, cattle, industry, and drinking water, we can expect an increase in the salinization of existing watersheds and water sources particularly in arid areas. These changes will negatively impact ecosystem processes [20]. Here we examine selected inland saline waters in the Chihuahuan Desert of México, a region renowned for its high biodiversity in terrestrial and aquatic systems [21-24]. We also present an initial species list of the rotifers, group sites by water chemistry, conduct pair-wise comparisons of rotifer community diversity between sites, and investigate ecological correlates of rotifer presence/absence.

## Results

### Water chemistries

**Figure 1:**
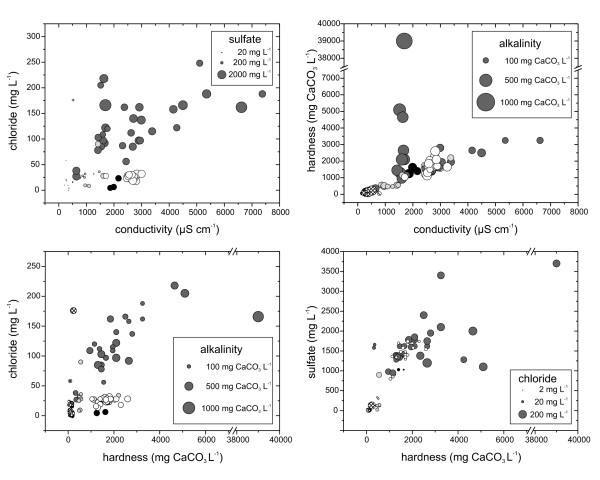
**Characterization of study sites by water chemistry.** Upper left panel - conductivity, chloride, and sulfate; upper right panel - conductivity, hardness, and alkalinity; lower left panel - hardness, chloride, and alkalinity; lower right panel - hardness, sulfate, and chloride. Sampling regions: black - San Luis Potosί; dark grey- Cuatro Ciénegas; white - Ojos Altos; light grey - Ojos en de Medio, de la Punta, de la Casa, Caliente (Camargo); cross hatched - Presa Chihuahua, Presa la Boquilla, Presa Francisco I. Madero, Lago Colina; hatched - Méxican spring flowing into Rio Grande downstream of Big Bend National Park (TX, USA).

**Figure 2:**
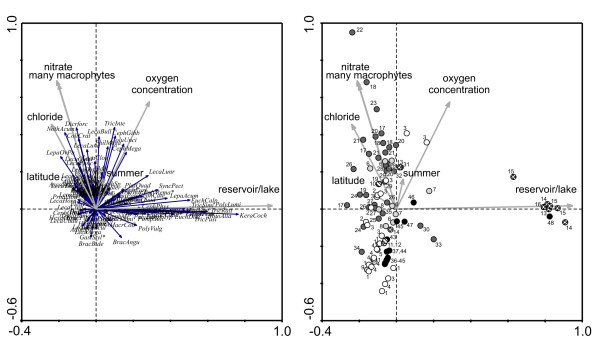
**RDA of all Méxican sites**. Left panel: ordination of species; Right panel: ordination of samples. Sampling regions: black – San Luis Potosí; dark grey – Cuatro Ciénegas; white – Ojos Altos; light grey – Ojos en Medio, de la Punta, de la Casa, Caliente (Camargo); cross hatched – Presa Chihuahua, Presa la Boquilla, Presa Francisco I. Madero, Lago Colina; hatched – Mexican site south of Big Bend National Park (TX, USA).

**Figure 3:**
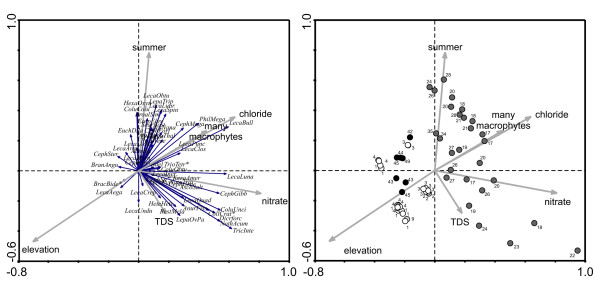
**RDA of Méxican sites with conductivity ≥2000 µS cm^-1^.** Left panel: ordination of species; * these species share a vector with other species: BracBide with PolyDoli, PolyVulg, GastStyl; CollCrat with CephPan, LecaCorn; TrioTetr with PlatQuad; Right panel: ordination of samples. Sampling regions: black - San Luis Potosί; dark grey - Cuatro Ciénegas; white - Ojos Altos; light grey - Ojo Caliente (Camargo).

**Figure 4:**
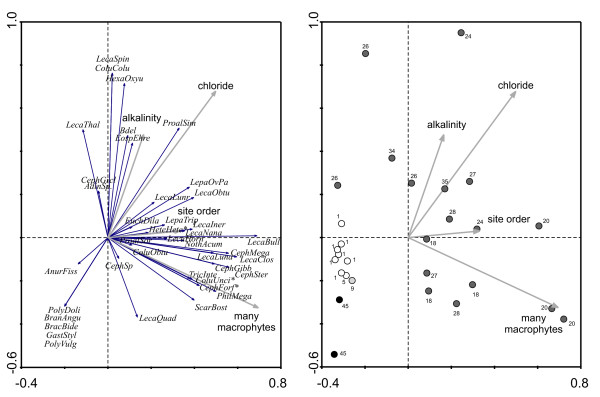
**RDA of Méxican sites with conductivity ≥3000 µS cm^-1^.** Left panel: ordination of species; * these species share a vector with other species: HeteHete with LecaCrep, LecaUndu; ColuUnci with TrioTetr, DicrForc, PlatQuad; CephForf with TripPlic, LecaArcu; Right panel: ordination of samples. Sampling regions: black - San Luis Potosί; dark grey - Cuatro Ciénegas; white - Ojos Altos; light grey - Ojo Caliente (Camargo).

With the exception of Cuatro Ciénegas (CC), sites in different regions cluster together as expected from their shared basins and geochemistries (Figs. 1, 2, 3, 4). The variety of water sources (springs, playas, rivers, seeps, wetlands) sampled at CC likely explains the spread in the data for these systems. In general these systems have higher conductivity, chloride, and sulfate than the others. Sulfate and chloride were positively associated with hardness in all sites. Alkalinity was less variable than other selected parameters.

### Site similarities in species richness

We found 57 species of monogonont rotifers in aquatic systems with salinities >2000 μS cm^-1^, and, of these, 34 also were present when salinities were ≥ 3000 μS cm^-1 ^(Table 1). In addition, we found many bdelloid species, but only *Philodina megalotrocha *was identified to species. For new records in México and total species count see [25]. Many of these species occurred as singletons (33.3%) or doubletons (21.7%), a feature that has been reported previously [25]. When comparing species composition between sites using the Simpson's Asymmetric similarity Indices (SAI), we found values ranging from 0.00 to 1.00. An SAI of 1.00 indicates complete unity of one site to the next, while SAI = 0.00 means complete dissimilarity of one site to the next (Table 2). The Jaccard Index also varied greatly among sites (i.e., 0.00 and 0.35, Table 3). In general, sites at Cuatro Ciénegas were quite distinct from those near Ciudad Juárez, MX. For example, disparity in SAI values between Ojos Altos A (site 1) and several sites at CC (sites 18–27) can be attributed to the paucity of species in the former and the richer fauna in the later (Table 2). A few sites had SAI values reflecting similarity in species composition: e.g., Rio Mesquites (site 18) and Las Playitas (site 27) and Los Hundidos (site 24) and Los Gatos (site 26) had reciprocal pairwise SAI values of approximate 0.5 (Table 2). Such similarity in rotifer fauna probably reflects the similarity in water chemistry of these sites, which is high in CaSO_4_.

**Table 1:** Species found in high salinity aquatic habitats in México (*found at salinities ≥ 3000 μS cm^-1^)

Number	Species	Abbreviation	Sites found
1	*Adineta *sp. *	AdinSp	34
2	Anuraeopsis fissa* Gosse, 1851	*AnurFiss*	22, 26, 45
3	*Ascomorpha saltans *Bartsch, 1870	*AscoSalt*	17, 21
4	Brachionus angularis* Gosse, 1851	*BracAngu*	42, 45
5	Brachionus bidentatus* Anderson, 1889	*BracBide*	45
6	*Cephalodella *sp.*	*CephSp*	27
7	Cephalodella forficula* (Ehrenberg, 1832)	*CephForf*	20, 21
8	Cephalodella gibba* (Ehrenberg, 1832)	*CephGibb*	18, 20, 22, 23, 27
9	Cephalodella gracilis* (Ehrenberg, 1832)	*CephGrcl*	3, 34
10	*Cephalodella *cf*. graciosa *Wulfert, 1951	*CephGrac*	21
11	Cephalodella megalocephala* (Glascott, 1893)	*CephMega*	17, 20, 23, 26, 27, 28
12	*Cephalodella panarista *Myers, 1924	*CephPana*	22
13	Cephalodella sterea* (Gosse, 1887)	*CephSter*	3, 4, 20
14	*Collotheca crateriformis *Offord, 1934	*CollCrat*	22
15	*Colurella colurus *(Ehrenberg, 1930)	*ColuColu*	24, 26
16	Colurella obtusa* (Gosse, 1886)	*ColuObtu*	3, 4, 17, 18, 34
17	*Colurella uncinata *(Müller, 1773)	*ColuUnci*	20, 21, 22
18	*Dicranophorus forcipatus *(O.F. Müller, 1786)	*DicrForc*	20, 23
19	Eosphora ehrenbergi* Weber & Montet, 1918	*EospEhre*	20, 26, 27
20	*Euchlanis dilatata *Ehrenberg, 1832	*EuchDila*	28, 44
21	Gastropus stylifer* (Imhof, 1891)	*GastStyl*	45
22	Hexarthra oxyuris* (Sernov, 1903)	*HexaOxyu*	24, 26, 35
23	*Keratella americana *Carlin, 1943	*KeraAmer*	17
24	*Lecane aeganea *Harring, 1914	*LecaAega*	4
25	Lecane arcula* Harring, 1914	*LecaArcu*	20, 43, 44
26	Lecane bulla* (Gosse, 1851)	*LecaBull*	3, 17, 18, 19, 20, 21, 22, 23, 24, 26, 27, 28, 34, 44
27	Lecane closterocerca* (Schmarda, 1859)	*LecaClos*	3, 20, 22, 34, 43
28	*Lecane cornuta *(O.F. Müller, 1786)	*LecaCorn*	22
29	*Lecane crepida *Harring, 1914	*LecaCrep*	24, 44
30	Lecane hornemanni* (Ehrenberg, 1834)	*LecaHorn*	3, 20, 34
31	Lecane inermis* (Bryce, 1892)	*LecaIner*	3, 21, 24
32	*Lecane leontina* (Turner, 1892)	LecaLeot	21, 44
33	Lecane luna* (O.F. Müller, 1776)	*LecaLuna*	17, 20, 21, 22, 23, 26, 35
34	Lecane lunaris* (Ehrenberg, 1832)	*LecaLunr*	3, 17, 18, 20, 26
35	Lecane nana* (Murray, 1913)	*LecaNana*	17, 20
36	Lecane obtusa* (Murray, 1913)	*LecaObtu*	20, 21, 26
37	*Lecane punctata *(Murray, 1913)	*LecaPunc*	17, 21
38	*Lecane pyriformis *(Daday, 1905)	*LecaPyri*	3, 17
39	Lecane quadridentata* (Ehrenberg, 1832)	*LecaQuad*	20, 22, 45
40	Lecane spinulifera* Edmondson, 1935	*LecaSpin*	21, 24, 26
41	*Lecane thalera *(Harring & Meyers, 1924)	*LecaThal*	3, 17, 19, 26
42	*Lepadella *(= Heterolepadella) heterostyla* (Murray, 1914)	*HeteHete*	24
43	Lecane undulata * Segers & Dumont, 1993	*LecaUndu*	3, 4, 24
44	Lepadella ovalis/patella* (O.F. Müller, 1786)	*LepaOvPa*	1, 2, 3, 4, 17,18, 19, 20, 21, 22, 23, 24, 26, 27, 35
45	Lepadella triptera* (Ehrenberg, 1830)	*LepaTrip*	17, 20, 21, 26
46	Notholca acuminata* (Ehrenberg, 1832)	*NothAcum*	20, 22, 23, 24, 26
47	*Notommata tripus *Ehrenberg, 1838	*NotoTrip*	21
48	Philodina megalotrocha* Ehrenberg, 1832	*PhilMega*	18, 20, 21, 28
49	*Platyias quadricornis *(Ehrenberg, 1832)	*PlatQuad*	20
50	Polyarthra dolichoptera* Idelson, 1925	*PolyDolic*	45
51	Polyarthra vulgaris* Carlin, 1943	*PolyVulg*	45
52	Proales similis* de Beauchamp, 1907	*ProaSimi*	20, 27, 24, 34
53	*Proales sordida *Gosse, 1886	*ProaSord*	18
54	*Proales *cf. *wesenbergi *Wulfert, 1960	*ProaWese*	21
55	*Resticula melandocus *(Gosse, 1887)	*RestMela*	23
56	Scaridium bostjani* Daems & Dumont, 1974	*ScarBost*	20, 28
57	*Trichocerca *cf. intermedia* (Stenroos, 1898)	*TricInte*	18, 20, 23
58	*Trichotria tetractis *(Ehrenberg, 1830)	*TricTetr*	20
59	*Tripleuchlanis plicata *(Levander, 1894)	*TripPlic*	20
60	Unidentified bdelloids*	Bdel	1, 2, 3, 4, 17, 18, 19, 20, 21, 23, 24, 26, 27, 28, 34, 43, 44

Locality codes: Ojos Altos A* (1), Ojos Altos B (2), Ojos Altos C (3), Ojos Altos D (4), Poza la Becerra (17), Rio Mesquites*(18), Poza Azul (19), Poza Tortugas* (20), Poza Churince (21), Entrance to Ejido El Venado (22), Tio Julio (23), Los Hundidos Main pool* (24), Los Gatos* (26), Las Playitas* (27), wetland north of Los Hundidos*(28), Poza Los Arcos* (34), La Campaña*(35), Rio la Lloviznosa (42), Manantial los Peroles (43), Manantial San Sebastian (44), San Francisco Cattle Tank*(45). Note: For complete list of species found in all Méxican sites sampled contact the corresponding author.

**Table 2:** Simpson's asymmetric percent similarity indices (SAI) for 10 selected sites* in the Méxican Chihuahuan Desert

Sites	1	18	20	24	26	27	28	34	35	45
1	--	1.00	1.00	1.00	1.00	1.00	0.50	0.50	0.50	0.00
18	0.22	--	0.78	0.33	0.38	0.44	0.33	0.22	0.11	0.00
20	0.07	0.26	--	0.19	0.37	0.23	0.19	0.15	0.04	0.04
24	0.17	0.25	0.42	--	0.58	0.33	0.17	0.17	0.17	0.00
26	0.07	0.21	0.67	0.47	--	0.33	0.14	0.07	0.13	0.07
27	0.25	0.50	0.86	0.50	0.63	--	0.38	0.25	0.13	0.00
28	0.17	0.50	0.83	0.33	0.40	0.50	--	0.17	0.00	0.00
34	0.14	0.29	0.57	0.29	0.14	0.29	0.14	--	0.00	0.00
35	0.50	0.50	0.50	1.00	1.00	0.50	0.00	0.00	--	0.00
45	0.00	0.00	0.14	0.00	0.14	0.00	0.00	0.00	0.00	--

* Site numbers are the same as reported in Table 1. Upper right half of table: SAI for the first versus the second site (e.g., 1 v. 2; 1 v. 3; 9 v. 10). Mean (± 1SD) = 0.30 (0.31). Lower left half of table: SAI for the second versus the first site (e.g., 2 v. 1; 3 v. 1; 10 v. 9). Mean = 0.32 (0.28).

**Table 3:** Jaccard Index for 10 selected sites* in the Méxican Chihuahuan Desert

Sites	1	18	20	24	26	27	28	34	35	45
1	--	0.22	0.07	0.17	0.07	0.25	0.14	0.13	0.33	0.00
18		--	0.24	0.17	0.16	0.31	0.25	0.14	0.10	0.00
20			--	0.15	0.31	0.22	0.18	0.13	0.04	0.03
24				--	0.35	0.25	0.13	0.12	0.17	0.00
26					--	0.28	0.12	0.05	0.13	0.05
27						--	0.27	0.15	0.11	0.00
28							--	0.08	0.00	0.00
34								--	0.00	0.00
35									--	0.00
45										--

* Site numbers are the same as reported in Table 1. The mean Jaccard Similarity Index for the same sites = 0.13 (0.10).

### Ecological correlations

In terms of ecological parameters, the first four canonical axes in the RDA of all Méxican sites explained 14.2% of the variance in the species data (Table 4). The most important environmental variable in the model was whether or not the habitats were reservoirs or large lakes. None of the saline sites sampled belonged to this habitat type (Fig. 2, right panel). Species assemblages found at sites other than lakes or reservoirs were ordinated with chloride concentration, nitrate concentration, oxygen concentration, and presence of many macrophytes. In general, most samples from CC were positively correlated with these variables, whereas samples from the Ojos Altos and San Luis Potosí were negatively correlated.

**Table 4:** Summary of RDA statistics for the first four axes of all Méxican sites

	Axis 1	Axis 2	Axis 3	Axis 4
Eigenvalues	0.048	0.042	0.029	0.023
Species-environment correlations	0.802	0.649	0.717	0.720
Cumulative percentage variance of species data	4.8	9.0	12.0	14.2
Cumulative percentage of species-environment relation	27.4	51.4	68.1	81.1

Rotifer species that were ordinated together with the variable lakes/reservoirs included many planktonic species, such as *Keratella cochlearis, Trichocerca rattus, T. similis, Polyarthra *cf. *luminosa, P. euryptera, P. vulgaris, P. remata, Asplanchna girodi, A. brightwellii, S. oblonga*, and *S. pectinata *(Fig. 2, left panel). Species that were positively associated with chloride are *Notholca acuminata, Dicranophorus forcipatus, Collotheca crateriformis*, *Cephalodella panarista, Lecane cornuta *and *Lepadella ovalis/patella*.

The RDA performed on the dataset of sites with conductivity ≥ 2000 μS cm^-1 ^separates species assemblages at CC from those found in the Ojos Altos and at San Luis Potosí, respectively (Fig. 3, right panel), with 21.2% of the variance in the species data being explained by the first four canonical axes (Table 5). Species assemblages of different sites at CC vary more than those at the other sampling regions and are ordinated along gradients formed by the presence of macrophytes and chloride concentration, nitrate concentration, seasonality (summer) and TDS. Species found mostly in the Ojos Altos and San Luis Potosí and negatively correlated with chloride are *L. aeganea, B. bidentatus, P. dolichoptera*, and *P. vulgaris *(Fig. 2, left panel). In CC sites a large group of species is ordinated with presence of macrophytes and chloride concentration: *L. bulla, Philodina megalotrocha, Cephalodella megalocephala, Lecane punctata *and *L. closterocerca*. Another group of species was correlated with seasonality (summer): *Lecane obtusa, L. lunaris, Lepadella triptera, Hexarthra oxyuris, Proalis similis, Eosphora ehrenbergi*, and *Euchlanis dilatata*.

**Table 5:** Summary of RDA statistics for the first four axes of Méxican sites with a conductivity ≥ 2000 μS cm^-1^

	Axis 1	Axis 2	Axis 3	Axis 4
Eigenvalues	0.102	0.057	0.028	0.025
Species-environment correlations	0.791	0.790	0.651	0.674
Cumulative percentage variance of species data	10.2	15.9	18.7	21.2
Cumulative percentage of species-environment relation	43.4	67.7	79.6	90.4

The RDA of the dataset containing the sites with conductivity ≥ 3000 μS cm^-1 ^again identified chloride, the presence of macrophytes, alkalinity, and site order, as the most important variables explaining the variance in the species data (Fig. 3). Site order signifies that the waterbody consisted of a series of pools that were ordered downstream from the spring source. In this analysis 30.4% of the total variance can be explained by the first four canonical axes (Table 6). Occurrence of *Lecane spinulifera, Colurella colurus, Hexarthra oxyuris, Eosphora ehrenbergi, Proales similis*, and bdelloids at CC sites was positively correlated with alkalinity and chloride concentration, whereas the majority of species correlated with presence of macrophytes included *Philodina megalotrocha, Cephalodella forficula, Colurella uncinata, Dicranophorus forcipatus, Lecane arcula, Platyias quadricornis, Scaridium bostjani*, *Trichocerca intermedia, Trichotria tetractis*, and *Tripleuchlanis plicata*. *Brachionus bidentatus, B. angularis, P. dolichoptera*, *P. vulgaris*, and *Gastropus stylifer *were found in San Francisco cattle tank (San Luis Potosí) and were negatively correlated with chloride.

**Table 6:** Summary of RDA statistics for the first four axes of Méxican sites with a conductivity ≥ 3000 μS cm^-1^

	Axis 1	Axis 2	Axis 3	Axis 4
Eigenvalues	0.142	0.091	0.041	0.029
Species-environment correlations	0.858	0.935	0.793	0.756
Cumulative percentage variance of species data	14.2	23.4	27.4	30.4
Cumulative percentage of species-environment relation	46.39	77.0	90.4	100.0

## Discussion

While zooplankton inhabiting saline aquatic habitats have received some attention worldwide (e.g., China [5], Spain [26-29], Canada [14,30], Western US [31,32], Africa [33-36], Japan [37], Australia [38-41], Arabia [42]), there is a genuine need for additional studies of rotifers in saline and marine environments of México [3]. A recent study noted that 74% of rotifer species in México (n = 42) were cosmopolitan, 5% were restricted to North America, 10% were tropical, and 4% were shared with Europe-Asia-Africa [43]. Most work on rotifers in saline habitats in México has been done by Sarma and his colleagues. For example, Sarma & Elías-Gutiérrez [44] found 31 rotifer species in their survey of an estuarine lagoon; 11 of which were found in our study (*Platyias quadricornis*, *Tripleuchlanis plicata, Colurella uncinata, Lepadella ovalis, Lecane bulla, L. closterocerca*, *L. hornemanni*, *L. luna*, *L. obtusa*, *L. pyriformis*, and *L. thalera*). In addition, Sarma *et al*. [3] reported 37 species of rotifers in Mecoacan, a brackish (5–35 ‰) lagoon located in Tabasco. Our survey of 48 waterbodies in the Mexican Chihuahuan desert shared few of these species (*Anuraeopsis fissa, Ascomorpha saltans, Brachionus angularis, Euchlanis dilatata*, and *Lecane bulla*) all of which have reportedly cosmopolitan distributions [45]. Another recent study [19] reported 128 taxa from 36 aquatic sites in southeastern México including some brackish habitats. Of these species, 23 (*A. fissa*, *B. bidentatus*, *C.obtusa*, *C. uncinata*, *E. dilatata*, *K. americana*, *L. arcula*, *L. bulla*, *L. closterocerca*, *L. cornuta*, *L. crepida*, *L. hornemanni*, *L. leontina*, *L. luna*, *L. lunaris*, *L. obtusa*, *L. spinulifera*, *L. thalera*, *L. triptera*, *Platyias quadricornis*, *Polyarthra dolichoptera*, *Scaridium bostjani *and *Tripleuchlanis plicata*) also were found in our survey.

In a larger study of freshwater habitats in the Méxican Chihuahuan Desert that includes the sites reported here, Wallace et al. [25] note that many species occurred as singletons or doubletons, and species inhabiting particular sites are quite unique. Here we further address community similarity among high salinity habitats using the SAI, and again the uniqueness of communities is apparent (Table 2). While our study adds substantially to the characterization of rotifer communities, clearly much more research needs to be accomplished if we are to develop a good understanding of the biogeography of rotifers in saline waters in North America.

The saline systems in the Mexican part of the Chihuahuan Desert have less total dissolved solids and lower conductivity than some of the waters of the Northern Chihuahuan Desert in the United States, notably those at White Sands National Monument, New Mexico and the Bottomless Lakes near Roswell, New Mexico. Only few species typical for saline waters were found over a wide range of aquatic habitats in the Chihuahuan Desert, such as *Proales similis*. However, a redundancy analysis performed on the data of all the saline systems that we have sampled in the Chihuahuan Desert, showed that the presence or absence of macrophytes is an important variable in determining species composition in all of these systems (unpublished data).

High salinity and conductivity levels can have major impacts on zooplankton community structure. A study in coastal lakes found that salinity level had significant impacts on zooplankton [46], leading the authors to predict that relatively small increases in salinity levels will cause reduced biodiversity of freshwater ecosystems. In a mesocosm experiment manipulating salinity, Hart *et al*. [31] found dramatic shifts in zooplankton community structure and shifts in the abundance of many species. As salinity increased, densities of the dominant rotifer species decreased and at the highest salinities 2 species were reduced to very low numbers. Shiel and his colleagues found that salinity was a significant, but site-specific, factor in determining rotifer community composition in rivers in the Lake Eyre Basin [41]. Saline systems had reduced species richness compared to their freshwater analogs (0–4 versus 0–31). In the Mexican saline systems studied here, as salinity increases the number of species found decreases substantially (Table 1; Fig 1, 2, 3). Chloride is a significant factor in determining the occurrence of rotifers. Some species are positively correlated with chloride content and others negatively associated (see Results, Fig 2a, 3a). It appears in our systems that typical planktonic freshwater species (e.g., *Asplanchna brightwellii*, *A. priodonta*, *K. americana*, *K. cochlearis*, and *Synchaeta pectinata*) are replaced by salinity tolerant species such as *Hexarthra oxyuris *and *Notholca acuminata*.

## Conclusion

Inland saline systems often harbor diverse and unique community assemblages. Unfortunately, human exploitation can be extremely disruptive to ecosystem processes and services provided by these important water sources. During our sampling efforts, several historical springs near Janos, México were dry. In addition, Ojo de la Casa has recently dried and Ojo en de Medio has dried and re-surfaced in the past year, probably due demands of a geothermal electrical plant for cooling water and agricultural and domestic uses. Increasing human population size and global climate change will only make this scenario more prevalent. Thus, it is imperative that governmental agencies establish policies that protect these fragile ecosystems [47].

## Methods

### Sampling strategy

As part of a larger study on Chihuahuan Desert waters [25,48-50] we sampled 48 sites in the Méxican portion of the Chihuahuan Desert. Sites included springs, cattle tanks, tinajas, rivers, reservoirs, and artificially constructed ponds. Some of these systems comprised multiple basins with varying degrees of inter-site connectivity. Of these, 11 sites had salinities ≥ 2000 μS cm^-1^, 10 additional sites had salinities from ≥ 3000 μS cm^-1^. It should be noted that sampling effort was not equal among all sites, some sites were sampled only once while others were sampled up to 7 times (Ojos Altos).

Our sampling strategy attempted to provide an All Taxa Biological Inventory (ATBI) [51]; to accomplish this we collected samples from planktonic, littoral, and benthic habitats using plankton nets (64 μm), grab samples (e.g., aquatic plants for sessile forms), and aspirating samplers. We calculated species richness (*S*), Jaccard's Similarity Index and Simpson's Index of Asymmetry [52,53]. The keys used in this study were as follows: Monogononta [54-64] and Bdelloidea [65,66]. Additional details of our methodology are described in [25,48,49].

### Analysis

To compare physical aspects of the aquatic habitats sampled, we constructed three-way plots of selected water chemistry parameters. To investigate ecological correlates of species distributions we conducted Redundancy Analyses (RDA) using CANOCO for Windows 4.54 [67]. Three RDAs were done: one using the complete dataset of the 48 sites sampled, a second analysis with a subset of data including the sites with conductivity >2000 μS cm^-1^, and a third on a subset of data with sites with conductivity ≥ 3000 μS cm^-1^. Environmental variables were sequentially added to the model of each analysis when they provided extra fit to the model at a significance level of p < 0.05. The significance of variables was determined with Monte Carlo tests running 9999 permutations.

## Competing interests

The authors declare that they have no competing interests.

## Authors' contributions

EJW, TS, RLW, JVRA, and RRM all participated in collecting trips and in species identifications. EJW drafted the manuscript. TS ran the statistical analyses (three-way plots of water chemistry and Redundancy Analyses). RLW calculated the Jaccard's and Simpson's Asymmetric Indices. All authors read and approved the final manuscript.
